# Intra-Articular AAV9 *α*-l-Iduronidase Gene Replacement in the Canine Model of Mucopolysaccharidosis Type I

**DOI:** 10.1155/2023/7419017

**Published:** 2023-09-14

**Authors:** Raymond Yu-Jeang Wang, Shih-Hsin Kan, Haoyue Zhang, Jodi D. Smith, Afshin Aminian, Elizabeth Snella, Jackie K. Jens, Sarah P. Young, Patricia I. Dickson, N. Matthew Ellinwood

**Affiliations:** 1 Division of Metabolic Disorders, CHOC Children’s Specialists, Orange 92868, USA; 2 Department of Pediatrics, University of California-Irvine School of Medicine, Orange 92868, USA; 3 CHOC Children’s Research Institute, Orange 92868, USA; 4 Biochemical Genetics Laboratory, Duke University Health System, Durham 27710, USA; 5 Department of Veterinary Pathology, Iowa State University, Ames 50011, USA; 6 Orthopedic Institute, CHOC Children’s Hospital, Orange 92868, USA; 7 Department of Animal Science, Iowa State University, Ames 50011, USA; 8 Department of Genetics, Development, And Cell Biology, Iowa State University, Ames 50011, USA; 9 Department of Veterinary Clinical Sciences, Iowa State University, Ames 50011, USA; 10 Department of Pediatrics, Duke University, Durham 27710, USA; 11 Division of Genetics and Genomic Medicine, Department of Pediatrics, Washington University School of Medicine in St. Louis, St. Louis 63130, USA

## Abstract

Mucopolysaccharidosis type I (MPS I), an inherited lysosomal storage disorder characterized by deficiency of *α*-l-iduronidase (IDUA) activity, causes multisystemic pathology due to sequelae of accumulated heparan and dermatan sulfates (HS and DS), the substrates of IDUA. Current treatments, though life-prolonging, inadequately address skeletal dysplasia and do not forestall progressive and painful degenerative joint disease. Previous studies demonstrated that intra-articular enzyme replacement cleared cellular lysosomal storage and reduced joint inflammation. Three nontolerized MPS I canines were studied to assess safety, efficacy, and durability of *IDUA* gene replacement therapy delivered via intra-articular injection. After baseline joint tissue biopsies, the right shoulder and stifle of each animal were injected in the intra-articular space with AAV9-*IDUA* and contralateral joints with AAV9-*eGFP*. Animals received either 5E11 or 5E12 vector genomes/joint. Necropsy was performed at 2- or 52-week postinjection. All animals tolerated injections without adverse effects. At two weeks, supraphysiologic IDUA enzyme activity was measured in AAV9-*IDUA*-treated but not AAV9-*eGFP*-treated synovium, with corresponding normalization of HS content and synoviocyte morphology. The AAV9-*IDUA-*treated cartilage had normal physiologic levels of IDUA enzyme, reduced but not normalized HS and DS levels compared to untreated MPS I cartilage, and healthy chondrocyte morphology. Liver *IDUA* transgene and IDUA enzyme activity were identified, as was serum IDUA activity which was 40% of wild-type serum enzyme activity. At 52-week postinjection, AAV9-*IDUA*-treated synovium and cartilage IDUA enzyme activity declined in both animals, corresponding to high tissue HS and DS levels and severe lysosomal storage. Liver and serum IDUA activity levels were undetectable. A dose-dependent serum anti-IDUA antibody response was observed which, together with loss of transgene with age, likely contributed to decline in tissue enzyme activity and treatment efficacy. Our study demonstrates successful proof-of-concept for intra-articular gene replacement therapy as a treatment for MPS-related joint dysplasia. Our observations suggest the possibility of multimodal gene replacement therapy to address multiple refractory manifestations of MPS I. Subsequent studies, in conjunction with immune tolerization and functional assessments of joint pathology, will investigate this possibility.

## 1. Introduction

The mucopolysaccharidoses (MPSs) are a group of twelve inborn errors of metabolism caused by deficiency of glycosaminoglycan- (GAG-) degrading enzymes with resultant GAG storage in lysosomes and tissues and progressive multisystemic dysfunction [1, 2]. Mucopolysaccharidosis type I (MPS I), often referred to by the eponyms Hurler, Hurler-Scheie, and Scheie syndromes depending upon degree of central nervous system (CNS) involvement and velocity of disease progression, results from a deficiency of the activity of *α*-l-iduronidase (IDUA) and results in tissue storage of the GAG species heparan sulfate (HS) and dermatan sulfate (DS). Across the MPS I disease spectrum, affected individuals experience varying degrees of upper airway obstruction, cardiac valve dysplasia, hepatosplenomegaly, skeletal dysplasia, and neurologic involvement [3].

The storage of GAG in chondrocytes triggers an abnormal proliferation in epiphyseal cartilage and disturbed cartilage matrix, with subsequent skeletal dysplasia manifesting as abnormally shaped vertebral bodies (kyphosis), shallow acetabulae, hip dysplasia, and genu valgum [4]. Joint synovium is also affected, becoming hypertrophic and inflamed. Clinically, these changes result in painful degenerative joint disease, which severely reduces mobility, independence, and quality of life [5].

Treatments for MPS I, either hematopoietic stem cell transplant (HSCT) or intravenous enzyme replacement therapy (ERT) with recombinant human IDUA (rhIDUA), have reduced disease burden and extended life expectancy of MPS I patients into adulthood [6, 7]. The unifying premise of each therapy is the provision of functional IDUA enzyme to affected IDUA-deficient tissue, degrading accumulated HS and DS [8]. Clearance of accumulated substrate improves cellular function and ideally prevents subsequent symptomatology.

However, both HSCT and ERT are imperfect treatments. Undergoing HSCT entails significant risks of immunocompromise, graft-versus-host disease, incomplete engraftment, and/or death. Pursuit of ERT entails weekly IV infusions for the patient’s entire lifetime and does not cross the blood-brain barrier (BBB) to any significant degree, thus rendering it incapable of treating the CNS disease of neuropathic MPS I. Neither HSCT nor ERT adequately treat orthopedic disease, ostensibly the consequence of negligible IDUA delivery and the continued GAG accumulation and pathology progression in joint tissues and chondrocytes [9, 10]. Treated MPS I patients suffer pain, immobility, and severely reduced quality of life due to progressive joint contractures, deformities, and arthritis [11, 12]. Surgical interventions are required to ameliorate these complications; due to their multisystem comorbid conditions, MPS I patients suffer increased perioperative morbidity and mortality [13]. Consequently, gene replacement therapy is an attractive option, as it is potentially a single-dose therapeutic providing long-term, durable transgene expression [14, 15].

In the canine model of MPS I, which recapitulates many aspects of human MPS I disease, we demonstrated that monthly intra-articular injections of rhIDUA were well tolerated and resulted in significant reduction of synoviocyte and chondrocyte lysosomal storage, with concomitant reduction of joint inflammatory macrophages [16]. However, repeated articular injection into multiple affected joints is not ideal for MPS I patients. Herein, we report on the safety and efficacy results of a proof-of-concept study utilizing adeno-associated virus (AAV) serotype 9-mediated canine *IDUA* (c*IDUA*; hereafter referred to as *IDUA*) gene replacement, delivered intra-articularly to the MPS I canine model system.

## 2. Materials and Methods

### 2.1. IACUC.

This study was conducted under compliance approvals obtained from the Iowa State University IACUC (protocols 12-04-5791-K and 2-18-8714-K) and Institutional Biosafety Committee (protocol 18-D-0004-A). All dogs used in the study were bred and maintained at Iowa State University in accordance with the USDA and NIH guidelines for the care of dogs. The MPS I dog colony was maintained on a mixed genetic background. Animals were housed together for enrichment and provided enrichment items. Their environment was set to have constant 72-degree F temperature, humidity, 12-hour/12-hour light: dark cycles, and air circulated 15 times an hour. Animals had continuous access to water and were fed *ad libitum*. Breedings were conducted by artificial insemination, crossing heterozygous females and heterozygous or MPS I-affected males. MPS I-affected animals were identified by IDUA enzymatic assay and polymerase chain reaction in whole blood [17].

### 2.2. Vector Prep.

The AAV9 vector contains a payload consisting of the chicken *β*-actin promoter, the cytomegalovirus immediate early enhancer, a codon-optimized *IDUA* or *eGFP* sequence, and the rabbit *β*-globin polyadenylation sequence. Vector was produced by triple transfection of HEK293T cells, purified on iodixanol gradients, and assessed for infectious capacity and endotoxin content as described by Wang, et al. [18].

### 2.3. Treatment.

**Figure 1:**
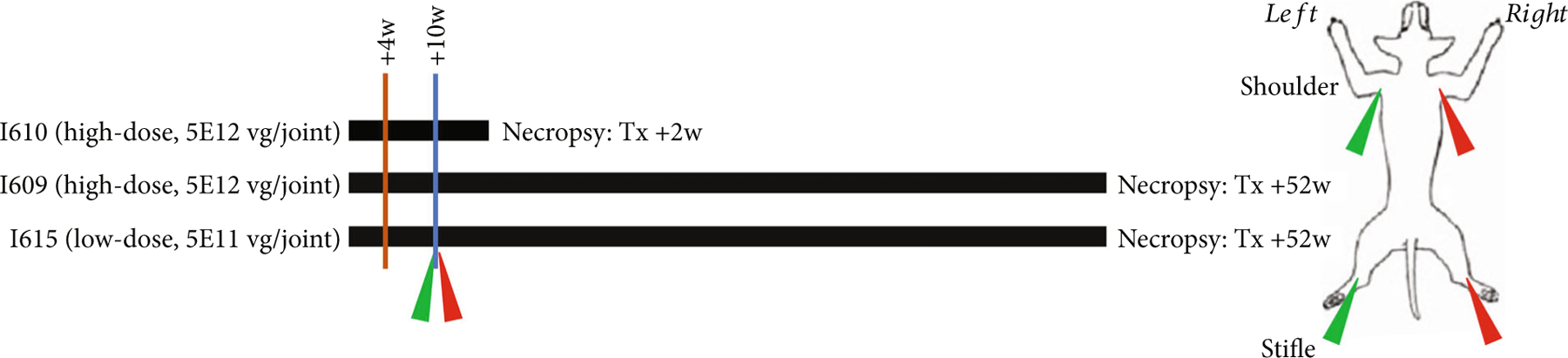
Study schematic. Four weeks after birth and six weeks prior to treatment, the three animals underwent pretreatment biopsy (brown vertical line). Treatment (blue vertical line) took place ten weeks after birth with dosages, vectors, and injection sites as designated. Necropsy took place +2 weeks after treatment (I610) or +52 weeks after treatment (I609 and I615). Green arrow indicates treatment with AAV9-*eGFP*; red arrow indicates treatment with AAV9-*IDUA*.

All animals received only intra-articular AAV vector and did not receive predose tolerization. Four weeks (see Safety) after baseline synovial and cartilage biopsies, one animal (low-dose +52 w) received 5E11 vg/joint AAV9-*IDUA* into the right shoulder and stifle and was necropsied 52-week postdosing. Four weeks following baseline biopsies, two animals received 5E12 vg/joint AAV9-*IDUA* into the right shoulders and stifles; one was euthanized 2 weeks following treatment (high-dose +2 w) and the other 52 weeks following treatment (high-dose +52 w). All three animals’ contralateral shoulder and stifle joints were injected with AAV9-green fluorescent protein (eGFP) at doses corresponding to their AAV9-*IDUA* dose. Please refer to Figure 1 for a schematic of the study. Following injection, monthly complete blood counts with differential, electrolytes, transaminases, blood urea nitrogen, creatinine, bilirubin, albumin, and protein were obtained.

### 2.4. Necropsy.

All three AAV-treated dogs were euthanized with intravenous sodium pentobarbital overdose at either 2- or 52-week posttreatment. All dogs were necropsied immediately following euthanasia. Aspiration of the shoulder and stifle joints was attempted for synovial fluid collection. Cartilage and synovium samples were collected from the proximal and distal surfaces of bilateral glenohumeral/shoulder and tibiofemoral/stifle joints. Samples were collected into 4% paraformaldehyde or flash frozen in liquid nitrogen. After 24 hours of fixation, samples for histology were processed by routine methods and embedded in paraffin. Serial 5 *μ*m thick sections were cut for histochemical staining. Tissues were obtained from three age-matched wild-type and three untreated MPS I-affected animals to be used as controls for biochemical assays and histology.

### 2.5. Histopathology and Scoring.

Histologic sections were deparaffinized in xylene and rehydrated through graded concentrations of ethanol. Sections were stained in hematoxylin and eosin (H&E), dehydrated, cleared, and mounted. All sections were prepared and examined by the same pathologist, who was blinded to animal identity and treatment status, using a Nikon Eclipse Ci brightfield microscope and photomicrographs acquired with a Nikon DS-Fi2 color CCD camera.

**Figure 2:**
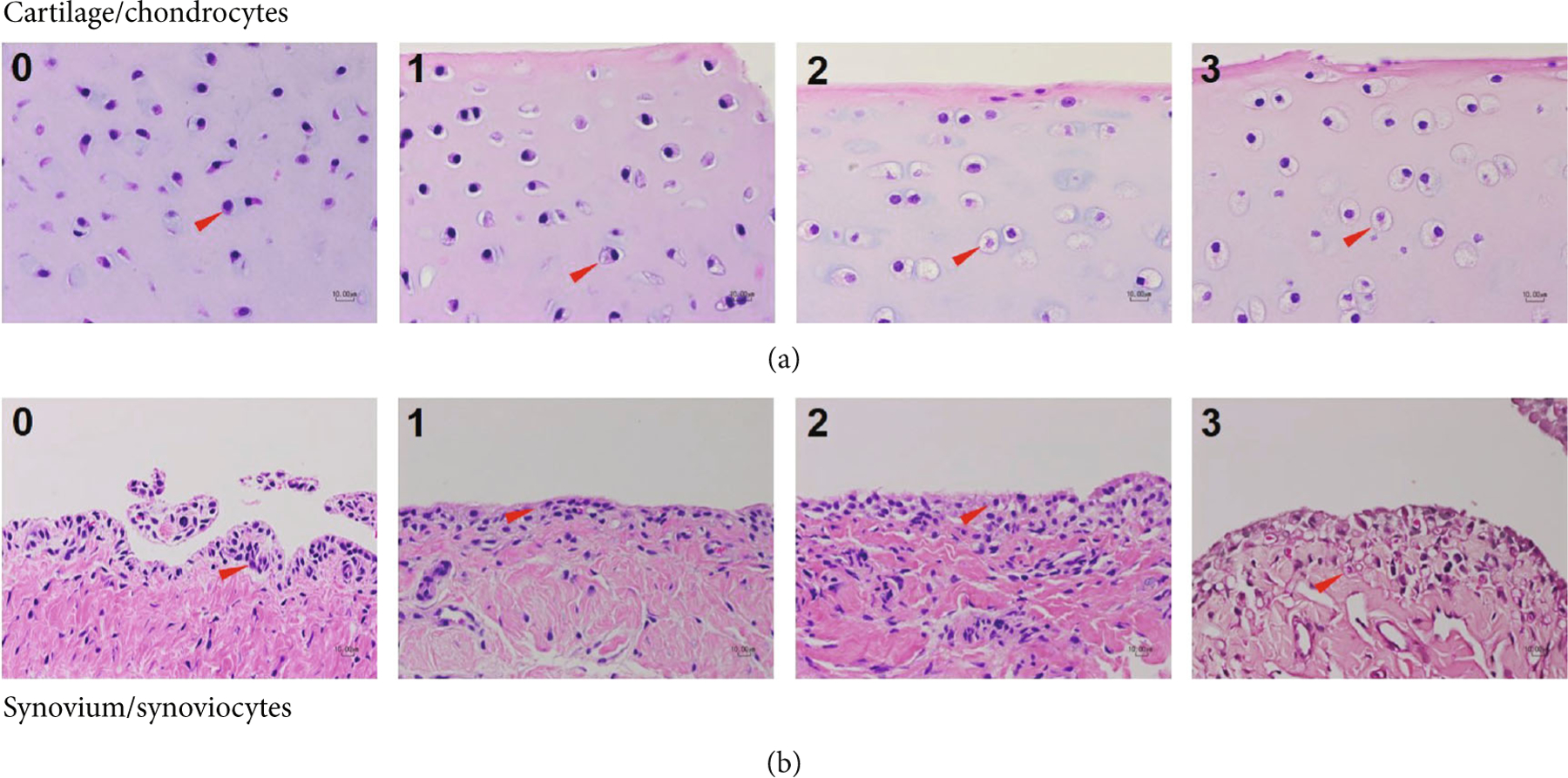
Exemplary joint tissue photomicrographs. Cartilage (a) and synovium (b) photomicrographs demonstrate scoring schema for lysosomal storage in chondrocytes and synoviocytes, respectively. Representative cells are denoted by red arrowheads.

Lysosomal distension of synoviocytes and chondrocytes was scored on a scale of 0–3 as follows: 0 signifies little to no storage (cytoplasm is devoid of vesicles); 1 signifies mild storage (cytoplasm with small number of vesicles); 2 signifies moderate storage (cell is predominantly vesicles with a small amount of cytoplasm); and 3 signifies severe (cell is entirely lysosomal vesicles) storage. Photomicrographs examples of synoviocytes and chondrocytes corresponding to each storage score are provided in Figure 2.

### 2.6. Enzymology.

For biochemical assays, canine tissues were homogenized in CelLytic M cell lysis reagent (Millipore-Sigma). Iduronidase activity was assessed using 10 *μ*L of tissue homogenate or serum incubated with 4-methylumbelliferyl *α*-l-iduronide substrate (4-MUI) (Bio-synth) at the final concentration of 0.25 mM for 1 hr at 37°C in a 96-well plate. Reactions were quenched with 180 *μ*L glycine carbonate buffer, and pH 10.5 and fluorescence measurements were obtained using an Infinite M Plex spectrofluorophotometer (Tecan) at excitation and emission wavelengths of 360 nm and 450 nm, respectively. One activity unit was defined as 1 nmol of 4-methylumbelliferone released per hour. Protein concentration was estimated using the Pierce BCA assay kit (ThermoFisher), and bovine serum albumin provided from the kit was served as a standard. Measurements were made blinded to identity of the animal and treatment administered, in triplicate, and reported as specific activity (units of activity per mg of protein) for tissue samples and units/mL for serum samples.

### 2.7. Tissue PCR Amplification.

Total genomic DNA was extracted from tissue homogenate by the Genomic DNA Clean and Concentrator (Zymo Research, Irvine, CA). The DNA samples were amplified with two sets of PCR primer pairs with the following sequences: *IDUA* F3 (5′-AGTTCCGGCGCATGCGCGCAGC-3′); *IDUA* R3 (5′-CGGTCGGGCCCAGTAGTCCACC-3′); *IDUA* F4 (5′-GTGGTGCTGAGGTCAGTCTCCG-3′); *IDUA* R4 (5′-CGGGCAGGGCCTGGGGTCCTAC-3′). The F3 and R3 primers are located within *IDUA* exon 10 and exon 14, respectively, and will amplify only transgenic *IDUA* cDNA sequences with an expected amplicon size of 389 bp; the F4 and R4 *IDUA* primers are located within *IDUA* intron 6 and intron 7, respectively, which amplify the endogenous *IDUA* gene with an expected amplicon size of 227 bp.

### 2.8. Glycosaminoglycan Quantification.

Lyophilized tissue extract was desalted using Amicon 3k columns (Millipore) and eluted with diH_2_O to the final concentration of 0.1 mg/*μ*L (tissue dry weight/H_2_O volume) before HS and DS analyses. Twenty-five *μ*L of tissue extracts, calibrators, and QC samples were transferred to a 12 × 32 mm maximum recovery vials (Waters Corporation, Milford, MA) and evaporated under nitrogen at 35°C. The samples were mixed with 200 *μ*L of 3 M HCl in MeOH, incubated at 65°C for 65 min, and evaporated under nitrogen at ambient temperature. The samples were reconstituted in 25 *μ*L of internal standard solution and 200 *μ*L of matrix (10 mM NH_4_OAc) in acetonitrile: H_2_O (90 : 10, *v* : *v*). The samples were injected on an Acquity UPLC^®^ I-class system coupled with a Xevo TQ-S micro mass spectrometer (Waters Corporation) with electron spray ionization in positive ion mode. Methylated HS and DS dimers and the corresponding deuteriomethylated HS and DS dimers were separated on a UPLC^®^ BEH Amide 1.7 *μ*m, 2.1× 50 mm column (Waters Corp.) using a linear gradient as previously described [19]. Data were acquired by selected reaction monitoring (SRM) using the protonated molecular ion transition m/z 426 → 236 for DS dimers, *m*/*z* 432 → 239 for ^2^H6-DS dimers, the sodiated molecular ion transition *m*/*z* 406 → 245 for HS dimers, and *m*/*z* 412 → 251 for ^2^H6-HS dimers. Calibration curves were constructed using linear regression of responses (peak area ratios of HS and DS dimers to the corresponding deuterium-labeled dimers) to the concentrations of calibrators using TargetLynx^®^ software (Waters Corp.) with 1/x weighting. Assessors were blinded to tissue type, genotype/treatment status of animal, and treatment administered.

### 2.9. Anti-rhIDUA Serology.

Serum and synovial fluid IgG antibodies against IDUA were measured by ELISA. Briefly, 96-well plates (Immulon 1B U-bottom microtiter plates, Corning, Corning, NY) were coated overnight with 0.2 *μ*g IDUA in PBS. Following blocking with 3% BSA, serial diluted serum samples were added in triplicate to IDUA-coated wells and incubated at 37°C for 1 hr. The plates were washed, and specific binding of serum anti-IDUA antibodies to the coated wells was detected using alkaline-phosphatase-conjugated goat anti-dog IgG secondary antibody (Southern Biotechnology, Birmingham, AL). Absorbance values at 405 nm were measured as optical density (OD) using an Infinite M Plex spectrofluorophotometer (Tecan) after 1 mg/mL p-nitrophenol phosphate substrate (Sigma-Aldrich, St. Louis, MO) was added and developed at room temperature. The antibody titer is defined as the highest dilution of each sample with OD value higher than the background control. The OD value for a sample was taken from dilutions within the linear signal range.

### 2.10. Statistical Methods.

Due to semiannual animal estrus, low litter size, and 25% rate of inheriting MPS I, no power calculation was performed. All animals identified to have MPS I were utilized for this study; an equal number (three) of treated MPS I, untreated MPS I, and wild-type animals were utilized. Results are reported as mean ± SD and as -fold of wild-type levels. Postnecropsy HS and DS levels are reported as quantitative values and as a percentage of baseline HS and DS levels, respectively.

## 3. Results

### 3.1. Safety.

All three animals tolerated anesthesia, baseline tissue biopsies, and AAV9 joint injections well, ambulating within 12 hours of surgery without discomfort or lameness. The low-dose +52 w animal experienced a right hock hygroma and joint effusion following tissue biopsy. The excess fluid was drained via needle puncture, and the joint capsule was allowed to heal completely, delaying vector injection by two weeks (six-week postbiopsy). For consistency, the other two animals were also treated with vector at six-week postbiopsy (Figure 1). None of the animals experienced clinically significant abnormalities in hematology or serum chemistries throughout the study. Only the high-dose +2 w animal yielded synovial fluid in the shoulder and stifle joints at baseline and necropsy; no inflammatory cells were noted at baseline. At necropsy, synovial fluid in all joints demonstrated evidence of mild to moderate inflammation (neutrophils and activated macrophages).

### 3.2. Efficacy

#### 3.2.1. Clinical Assessments.

At the time of necropsy, both low-dose +52 w and high-dose +52 w animals displayed facial dysmorphism and joint laxity characteristic of canines with MPS I.

#### 3.2.2. Serum and Liver Treatment Effects.

At necropsy, the high-dose +2 w animal had serum IDUA enzyme activity measured at 2.16 ± 0.27 units/mL, which was 49.5% of mean wild-type serum IDUA (4.36 ± 1.31 units/mL). Unfortunately, we do not have corresponding serum blood draws for either the low-dose +52 w or high-dose +52 w animals at the same time point (two-week posttreatment). Serum enzyme activity levels in these two animals were measurable but quite low in both animals at 8-, 13-, and 22-week post-treatment but declined to zero by the time of necropsy (see Table 1).

**Figure 3:**
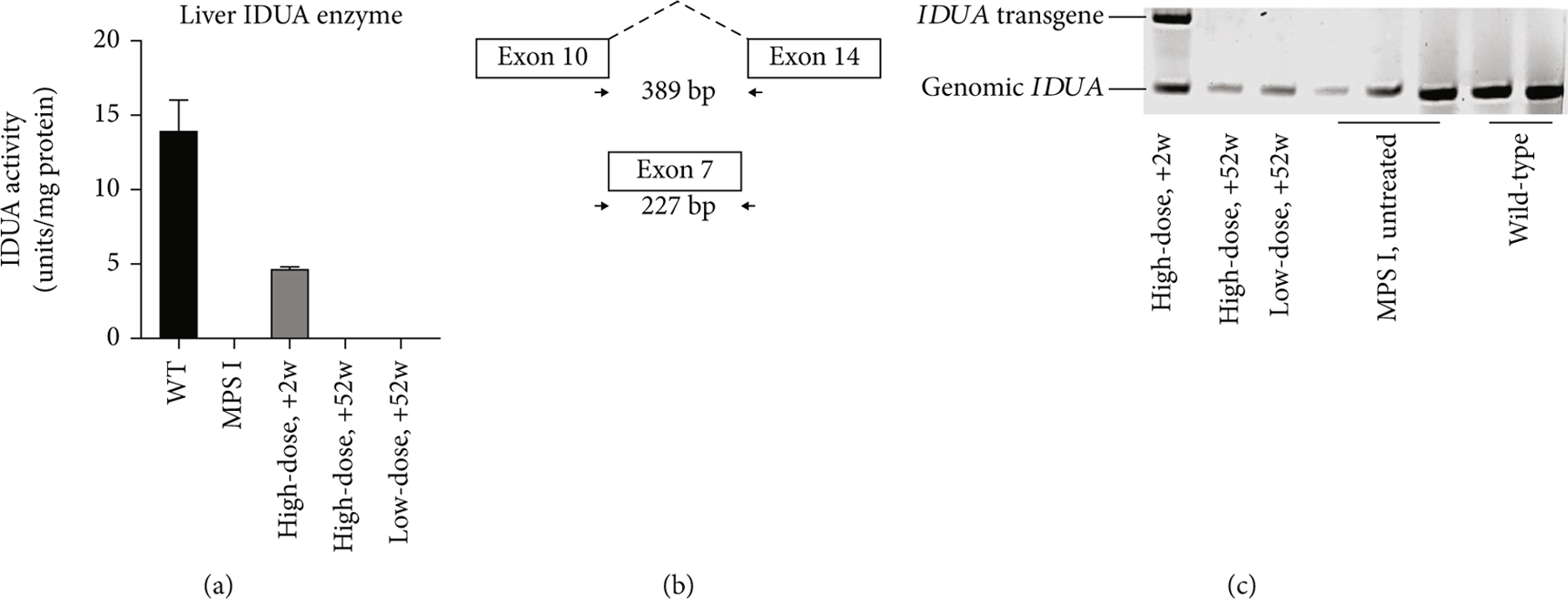
(a) IDUA enzyme activity measurements from homogenates from wild-type, MPS-I untreated, and study animals. Data were generated from at least three independent experiments and shown as mean ± SD. (b) Schematic of PCR primers utilized to detect the *IDUA* transgene (389 base pairs spanning cDNA between exon 10 and exon 14) and the native genomic *IDUA* gene (227 bp covering the entirety of exon 7). (c) Transgene and genomic IDUA PCR of liver homogenates. The presence of the native *IDUA* gene is identified in all animals, but only the high-dose +2 w dog demonstrates the presence of *IDUA* transgene and appreciable levels of IDUA enzyme in liver. The two +52 w animals had neither measurable liver IDUA enzyme activity nor *IDUA* transgene.

The liver of the high-dose +2 w dog had IDUA enzyme activity measured at 1.60 ± 0.11 units/mg protein, which was 34.8% of wild-type liver homogenate) and the presence of the *IDUA* transgene was evident by PCR. These findings suggest that the probable source of serum IDUA enzyme was the liver and not from leakage of IDUA enzyme from transduced joint tissues into the circulation. The low-dose +52 w and the high-dose +52 w animals demonstrated minimal IDUA enzyme activity and undetectable transgene in liver homogenates, indicating that transgene and expression declined significantly over time (see Figure 3). However, these findings raise the possibility that the liver can serve as an “IDUA depot,” synthesizing enzyme for other body tissues.

#### 3.2.3. Synovium Treatment Effects

##### (1) IDUA Expression and Enzyme Activity.

**Figure 4:**
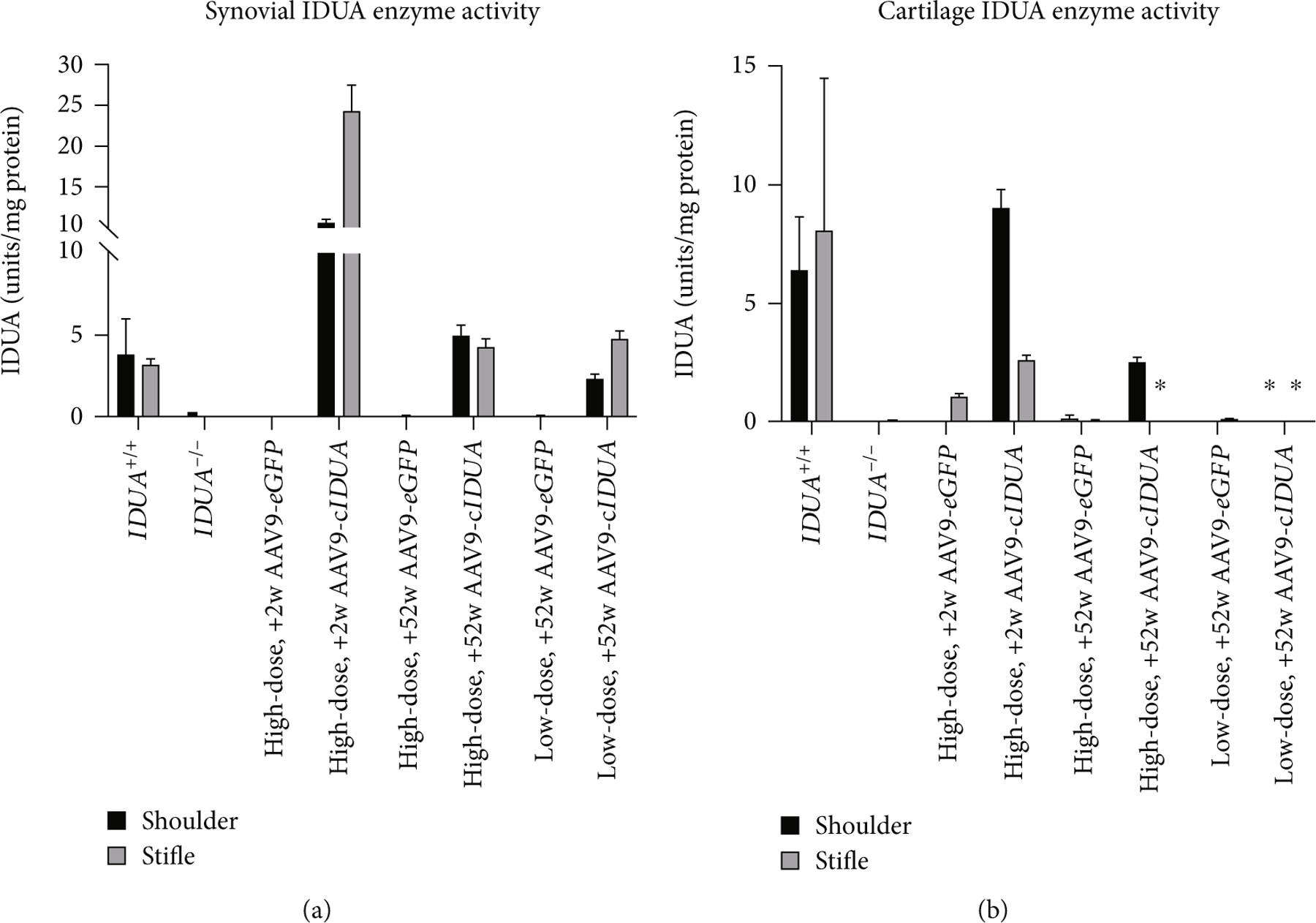
Joint tissue IDUA enzyme activity measurements. At 2 weeks, supraphysiologic enzyme levels are synthesized in AAV9-*IDUA*-treated synovium (a) (note *y*-axis) and physiologic levels in cartilage (b). AAV9-eGFP-treated tissues demonstrate undetectable to trace levels of IDUA enzyme. Synovial and cartilage IDUA levels are present in 52-week AAV9-*IDUA*-treated tissues. Data were generated from at least three independent experiments and shown as mean ± SD. ^∗^ could not be assayed.

The AAV9-*IDUA*-treated joints of the high-dose +2 w animal had synovial IDUA enzyme activity levels which were 2.97–6.91 fold that of wild-type dog synovium (3.50 ± 1.59 units/mg protein). Only the right shoulder synovium of this animal (IDUA activity, 10.44 ± 0.59 units/mg protein) had detectable *IDUA* transgene, while the right stifle synovium (IDUA activity, 24.20 ± 3.35 units/mg protein) had no measurable *IDUA* transgene signal (see Figure 1S). The high-dose +52 w (shoulder: 4.97 ± 0.66 units/mg protein, 1.42-fold wild-type; stifle: 4.23 ± 0.56 units/mg protein, 1.2-fold wild-type) and low-dose +52 w (shoulder: 2.27 ± 0.34 units/mg protein, 0.65-fold wild-type; stifle: 4.78 ± 0.49 units/mg protein, 1.36-fold wild-type) animals both had synovial IDUA enzyme activity in AAV9-*IDUA*-treated joints measured at levels comparable to wild-type dogs (see Figure 4). The *IDUA* transgene was not detected in any of the AAV9-*eGFP*-treated joints, which also had IDUA enzyme activity levels that were comparable to that of the synovium measured in untreated MPS I dogs.

##### (2) Synovial GAG Quantitation.

**Figure 5:**
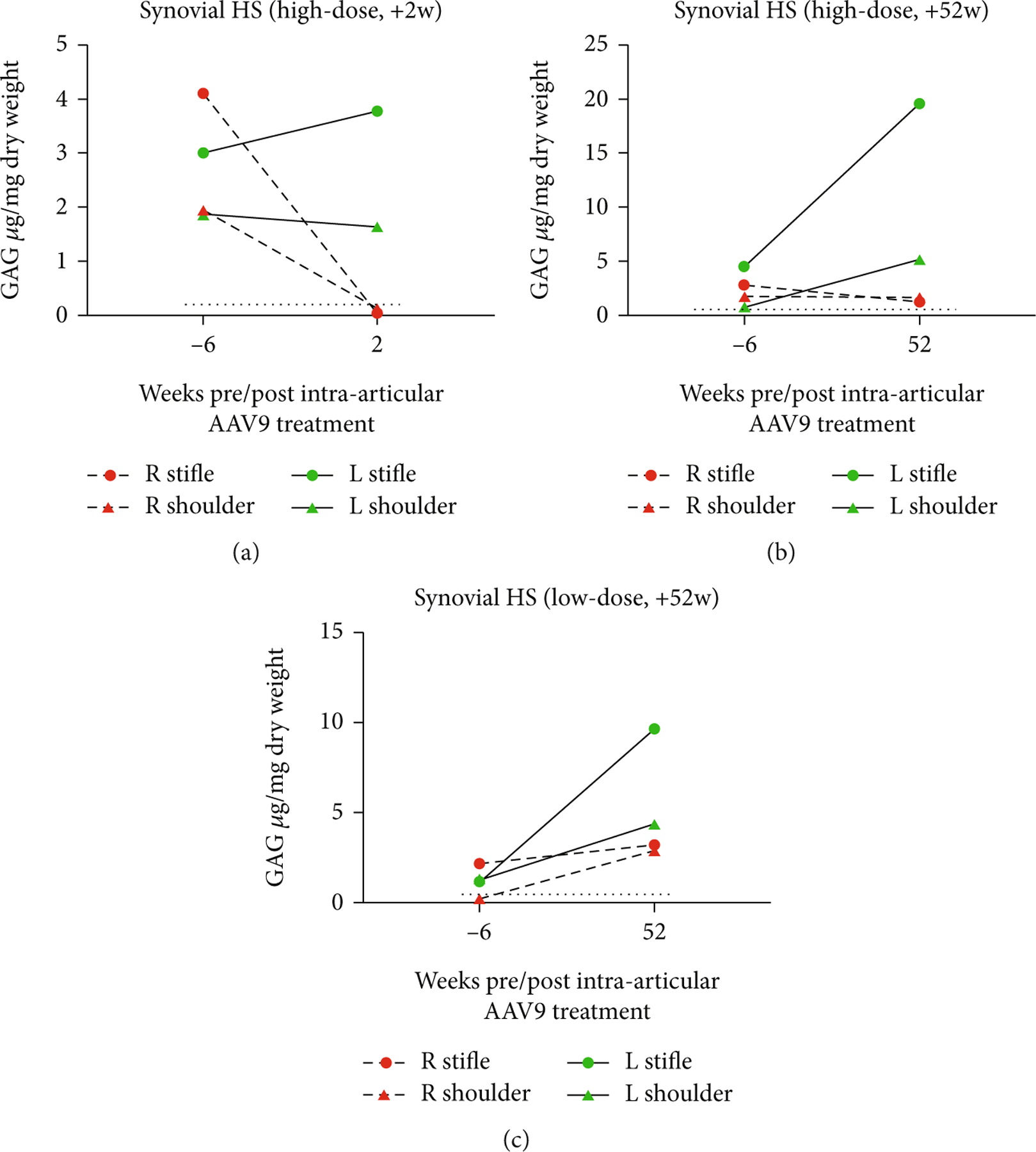
Synovial HS levels in the (a) high-dose +2 w, (b) high-dose +52 w, and (c) low-dose +52 w animals. Levels of synovial HS GAG at baseline (−6 weeks prior to AAV9 treatment) compared to levels at necropsy (+2- or +52-week post-AAV9 treatment). AAV9-*IDUA* joints are denoted in green, while AAV9-*eGFP* joints are denoted in red. The black dotted lines refer to synovial HS levels in age-matched normal dogs.

The HS and DS levels measured in synovial tissue of AAV9-*IDUA*-treated joints were decreased relative to untreated joints. Please refer to Table 2 for depiction of the data in chart format. In all animals, only AAV9-*IDUA*-treated joints demonstrate HS reduction. The greatest effect is seen in the high-dose +2 w animal, whose synovial HS levels are comparable to wild-type synovium. The high-dose +52 w treated animal demonstrated synovial HS at near-normal levels, while the low-dose +52 w animal demonstrated synovial HS above levels seen in wild-type and increased slightly from baseline. Meanwhile, all AAV9-*eGFP*-treated joints demonstrated higher HS levels than AAV9-*IDUA*-treated joints (see Figure 5).

**Figure 6:**
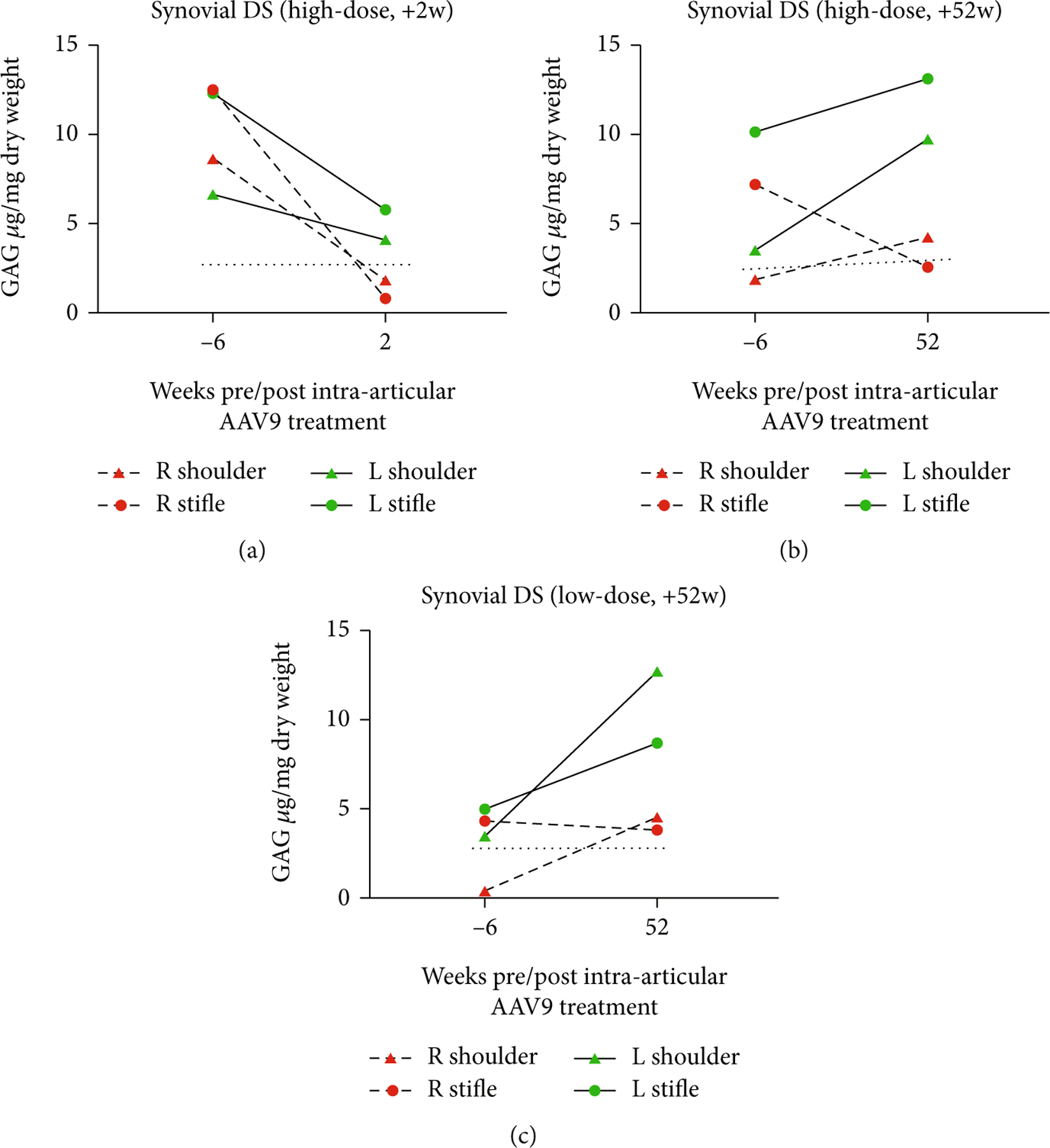
Synovial DS levels in the (a) high-dose +2 w, (b) high-dose +52 w, and (c) low-dose +52 w animals. Levels of synovial DS GAG at baseline compared to levels at necropsy. AAV9-*IDUA* joints in all three animals demonstrate synovial DS reduction, while AAV9-*eGFP*-treated joints show increases in DS levels in the two +52 w animals. The etiology for the DS reduction in AAV9-*eGFP*-treated joints in the +2 w-treated animal is unclear as its synovium has no measurable IDUA enzyme.

The measured levels of DS were similar to that of HS. Only AAV9-*IDUA* joints demonstrate DS reduction. The greatest effect is seen in the high-dose +2 w animal, whose AAV9-*IDUA*-treated synovial DS levels are comparable to wild-type synovial DS levels. Both +52 w-treated animals showed AAV9-*IDUA* synovial DS levels that were comparable to, or slightly above, normal animals, while DS levels in the AAV9-*eGFP*-treated joints increased over time (see Figure 6).

##### (3) Synovial Histopathology.

**Figure 7:**
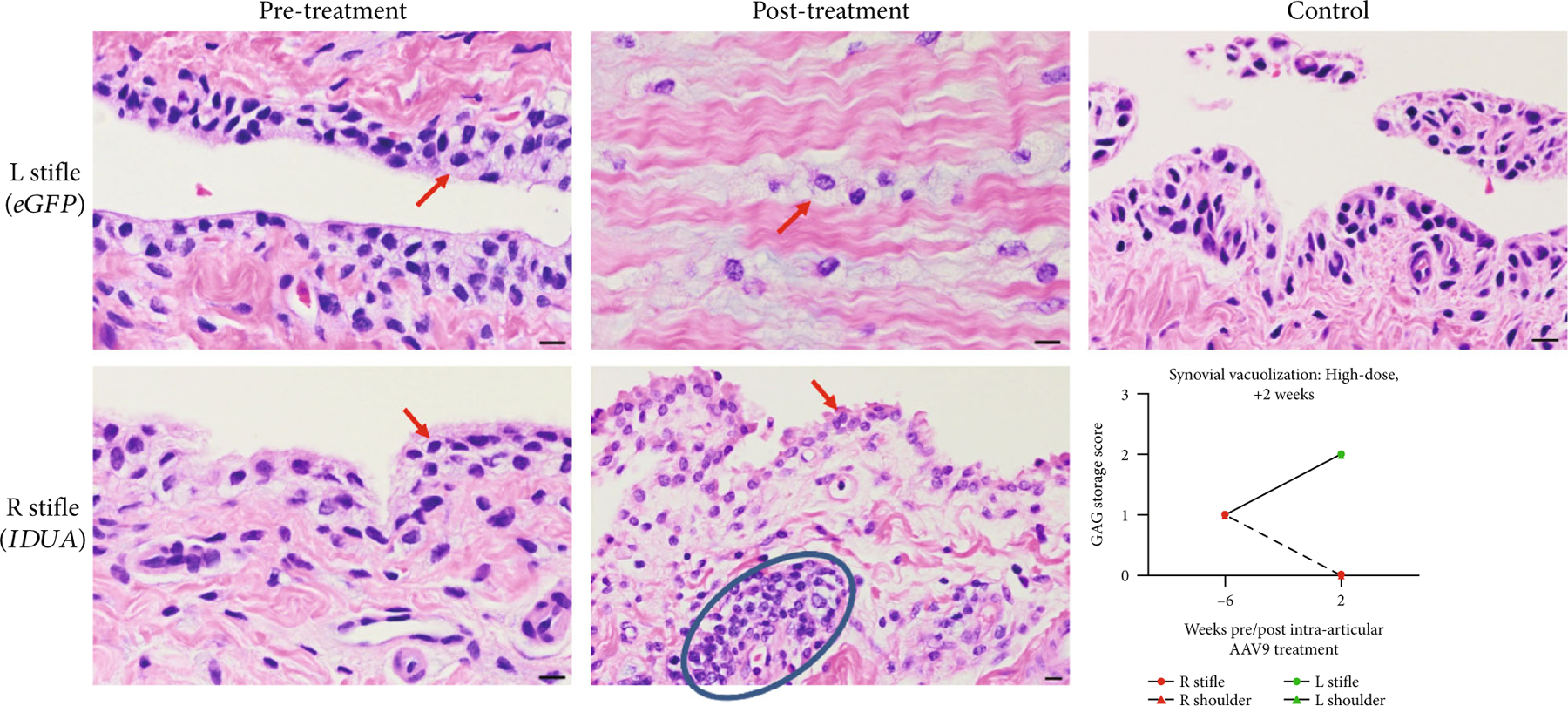
Synovial photomicrographs and lysosomal storage scoring in the high-dose +2 w animal. Baseline synoviocytes demonstrate lysosomal storage (red arrows, scored 1). At necropsy, AAV9-*eGFP* treated joint capsule showed increased lysosomal storage (scored 2), while AAV9-*IDUA*-treated synoviocytes scored 0. The latter also demonstrated a lymphocytic infiltrate (blue ellipse). Control synovium included for comparison. Bar = 10 *μ*M.

In the high-dose +2 w animal, histology examination of necropsy samples documented normal synoviocyte morphology with no visible synovial lysosomal storage (scored 0). Contralateral AAV9-eGFP treated tissue yielded joint capsule, not synovium. Capsular cells were documented to have abundant intracellular storage granules (scored 2). Lymphocytic infiltrates were noted only in AAV9-*IDUA-*treated synovium, not AAV9-*eGFP-* treated synovium (Figure 7). The scores of synovium from animals treated for 52 weeks were consistent with a dose-response, with the high-dose +52 w animal scoring 1 in AAV9-*IDUA* joints (eGFP joints scored 2) and low-dose +52 w animal scoring 3 in AAV9-*IDUA* joints (eGFP joints scored 3) (Figure 2S).

#### 3.2.4. Cartilage Treatment Effects

##### (1) IDUA Enzyme Activity.

The high-dose +2-week animal had cartilage IDUA enzyme activity levels which were 0.36–1.25-fold that of wild-type cartilage (7.25 ± 4.61 units/mg protein). Specifically, right shoulder cartilage demonstrated IDUA activity of 9.03 ± 0.76 units/mg protein, and right stifle cartilage showed IDUA activity of 2.59 ± 0.21 units/mg protein. Cartilage IDUA enzyme activity was 2.52 ± 0.20 units/mg protein in the right shoulder of the high-dose +52-week animal (0.35-fold wild-type). With the exception of stifle cartilage in the high-dose +2 w pup (15% of wild-type), only very low (<1.7% of wild-type) IDUA activity was detected in AAV9-*eGFP*-treated cartilage (see Figure 4).

##### (2) Cartilage GAG Quantitation.

AAV9-*IDUA* resulted in treatment effects for both HS and DS in cartilage. Please refer to Table 3 for depiction of the data in chart format.

**Figure 8:**
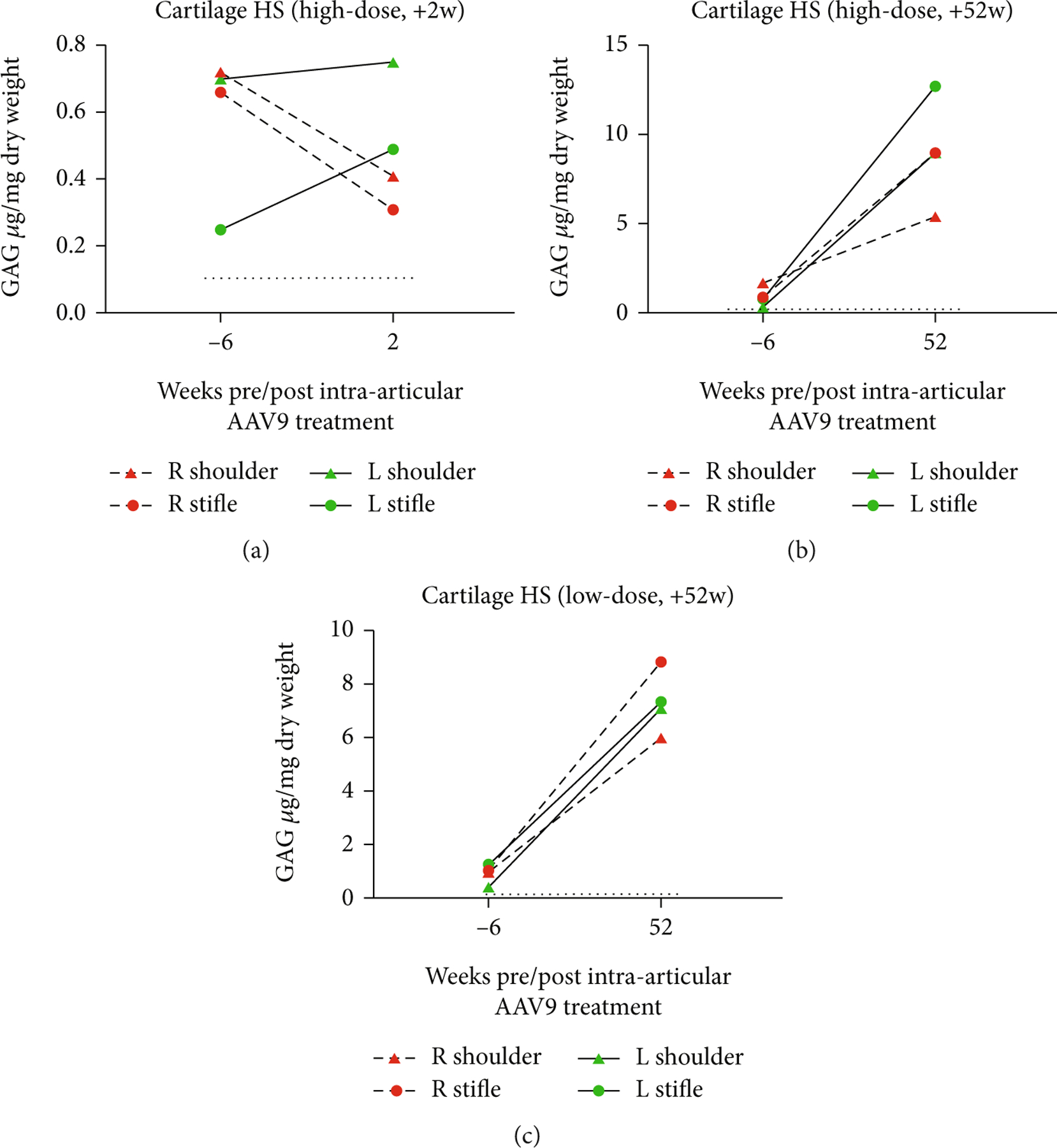
Cartilage HS levels in the (a) high-dose +2 w, (b) high-dose +52 w, and (c) low-dose +52 w animals. HS is only reduced in AAV9-*IDUA*-treated cartilage of the high-dose +2 w dog. In all other joints, cartilage HS was higher at necropsy than at baseline.

Only the AAV9-*IDUA*-treated joints of the high-dose +2 w animal demonstrated reduction of cartilage HS, which was still higher than wild-type control cartilage HS. The AAV9-*IDUA*-treated cartilage of the high-dose +52 w animal had lower HS levels relative to contralateral AAV9-*eGFP-*treated joints, but this level was still markedly above that of normal cartilage. The low-dose +52 w animal had cartilage HS levels measured at the level of an affected joint (see Figure 8).

**Figure 9:**
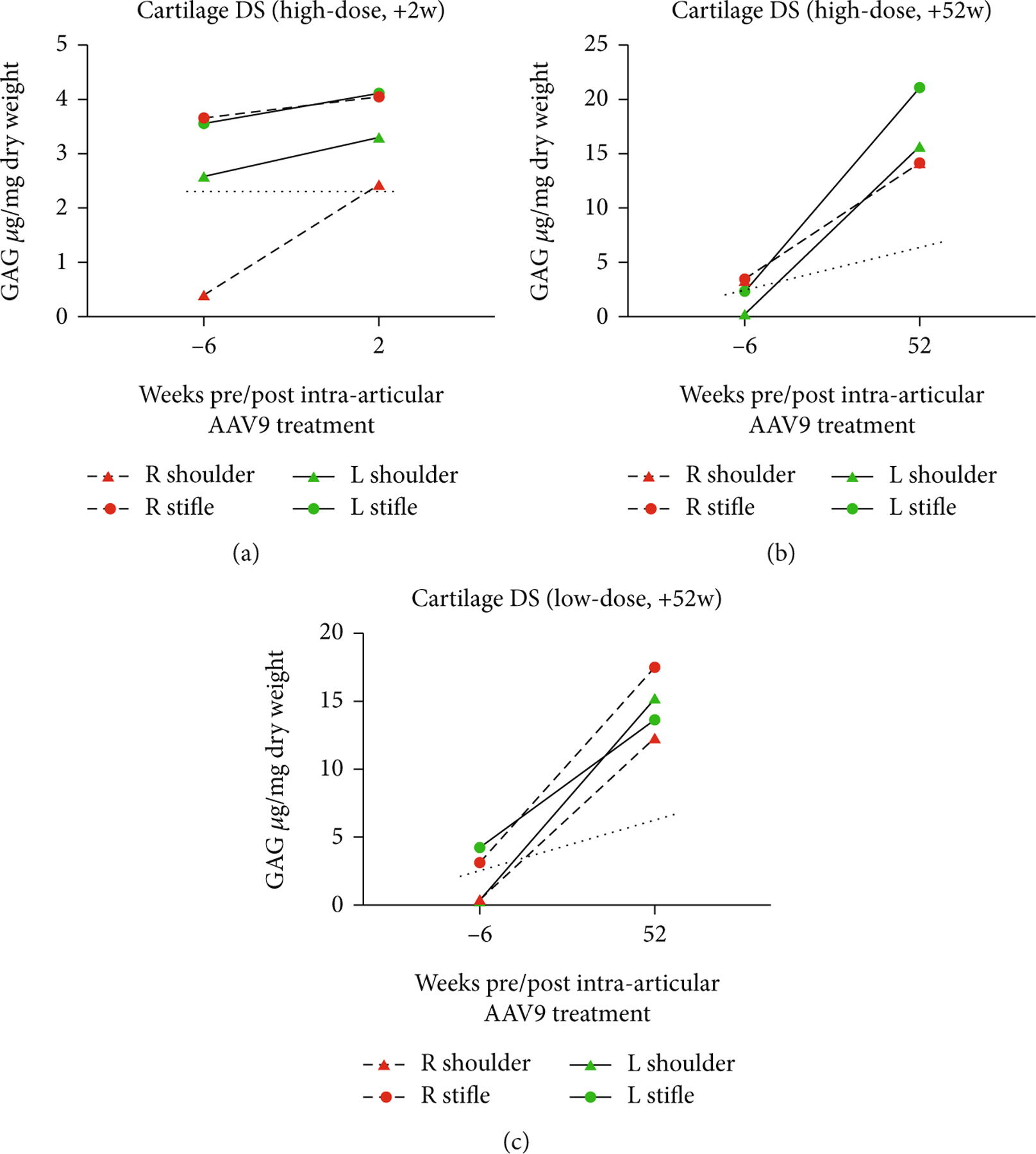
Cartilage DS levels in the (a) high-dose +2 w, (b) high-dose +52 w, and (c) low-dose +52 w animals. Cartilage DS levels in all treated animals were greater at necropsy than baseline. Only DS from the AAV9-*IDUA* shoulder of the high-dose +2 w animal was comparable to wild-type cartilage.

In all animals, cartilage DS increased from baseline to necropsy. The AAV9-*IDUA*-treated cartilage of the high-dose +2 w animal had DS levels that were lower relative to the contralateral AAV9-*eGFP*-treated joints but that were still elevated above wild-type cartilage DS levels. Both the high-dose +52 w and low-dose +52 w animals’ cartilage DS levels were increased relative to baseline (see Figure 9).

##### (3) Chondrocyte Histopathology.

**Figure 10:**
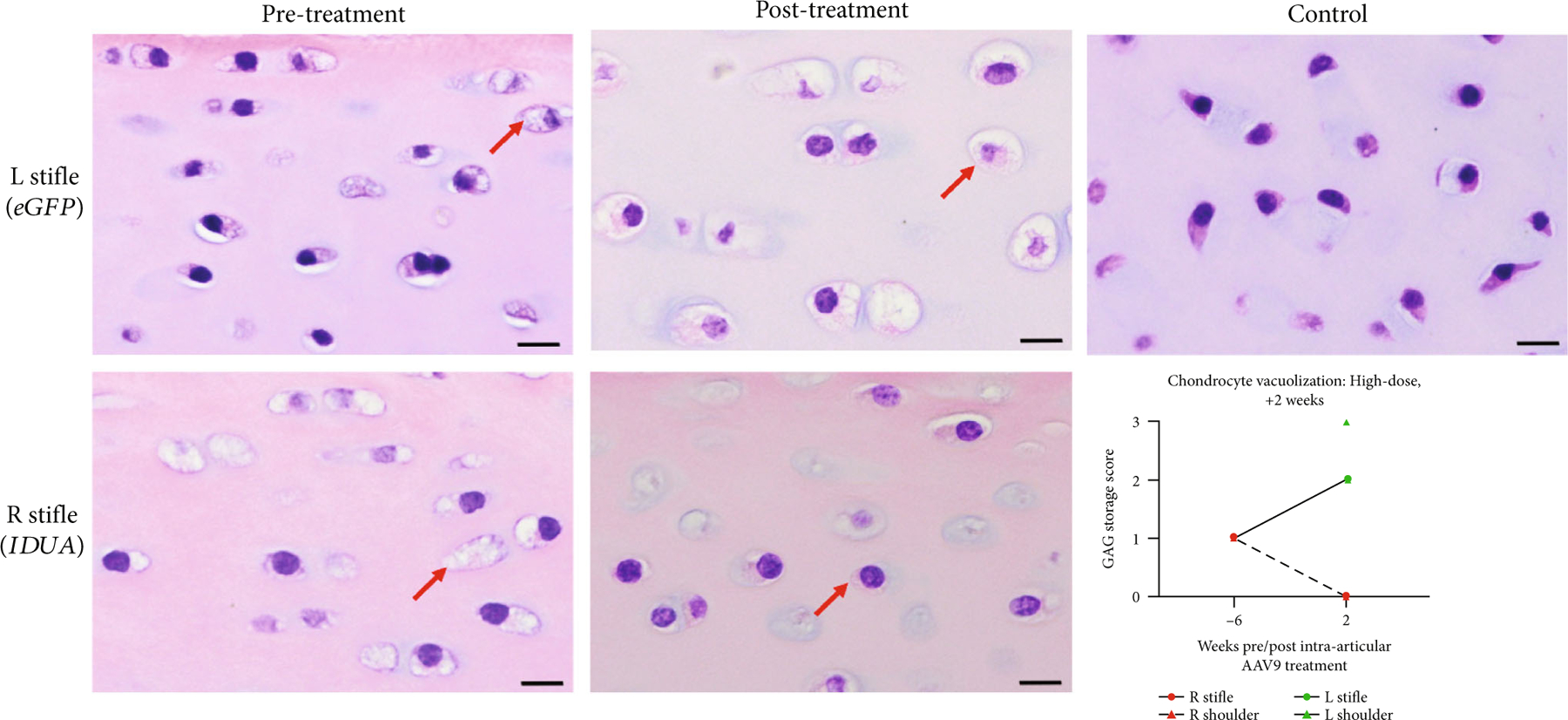
Cartilage photomicrographs and chondrocyte lysosomal storage scoring in the high-dose +2 w animal. At baseline, chondrocytes demonstrate foamy lysosomal storage (red arrows, scored 1). At necropsy, AAV9-*eGFP*-treated chondrocytes had increased lysosomal storage (scored 2 or 3), while AAV9-*IDUA*-treated chondrocytes demonstrated normal-appearing basophilic cytoplasm (scored 0). Control chondrocytes included for comparison. Bar = 10 *μ*M.

Only in the high-dose +2 w animal was there a clearance of chondrocyte lysosomal storage from baseline (Figure 10). In the two +52 w animals, all joint chondrocytes, regardless of treatment status, were documented to have severe vacuolization and lysosomal storage granules (Figure 3S).

#### 3.2.5. Immunologic Assays.

**Figure 11:**
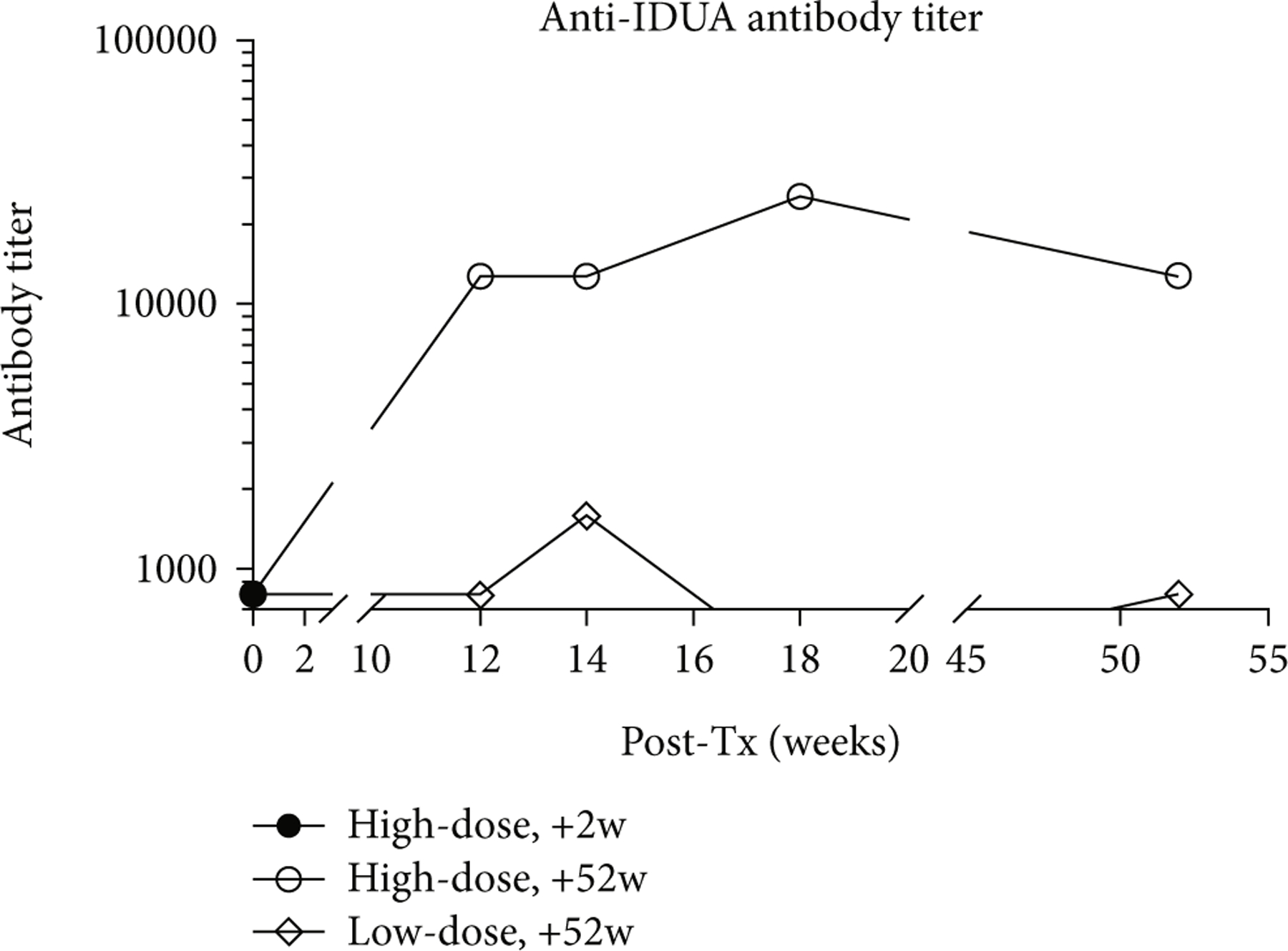
Serum anti-IDUA antibody levels. All animals had no anti-IDUA antibody at baseline. The high-dose +52 w animal demonstrated extremely high anti-IDUA serologies, while the low-dose +52 w animal did have detectable but very low anti-IDUA antibody levels.

No animal had serum anti-IDUA antibodies present at baseline. The results of anti-IDUA antibody ELISA conducted on necropsy sera identified the highest anti-IDUA IgG titers in serum of the high-dose +52 w animal (1 : 25,600 dilution). The low-dose +52 w animal developed a low serum anti-IDUA titer (1 : 800 dilution), and the high-dose +2 w animal had no measurable serum anti-IDUA antibodies, likely due to the short period of time between vector injection and necropsy (Figure 11). Synovial anti-IDUA antibodies were weakly positive (1 : 300) in all joints of the high-dose +2 w animal. The only synovial fluid sample available in the +52 w animals was the high-dose left (AAV9-*eGFP*) stifle with a moderately positive (1 : 2,700) titer.

## 4. Conclusion

Effective therapy for MPS-related arthropathy has been elusive; neither standard-of-care intravenous ERT nor HSCT deliver sufficient IDUA enzyme activity to joint synovium or cartilage for adequate tissue GAG clearance. Preclinical work in canine MPS I and VII have documented that supraphysiologic blood IDUA activity levels are required for therapeutic effect in difficult-to-treat areas such as cardiac valves and articular cartilage [20, 21]. Human MPS I clinical trial results corroborate this finding. Recent reports of treatment with autologous lentiviral-IDUA-transduced hematopoietic stem/progenitor cell transplant yielded blood IDUA activity levels 2.7- to 12.5-fold above median normal levels and gradual improvement in shoulder and knee joint range of motion 6–9 months posttransplant [22].

We aimed to assess whether intra-articular AAV *IDUA* gene replacement therapy could affect high-level joint IDUA expression, tissue GAG reduction, and ameliorate MPS I arthropathy. Intra-articular AAV-based therapies have been tested preclinically, primarily for osteoarthritis or rheumatoid arthritis indications in small and large animal model systems; several are currently in human clinical trials. One intra-articular nonviral gene replacement therapy has been reported for MPS I mice, employing IDUA plasmid nanoemulsions which resulted in supraphysiologic synovial fluid IDUA enzyme levels 48 hours posttreatment [23]. As the nanoemulsion method only expressed enzyme for up to 7 days, intra-articular gene replacement using viral vectors, which for intra-venous delivery results in longer-term transgene expression, represented a potentially more viable therapeutic approach for MPS I arthropathy.

Our study documents that AAV9-*IDUA* intra-articular injections at the 5E11 and 5E12 vg/joint dosages were safe and well tolerated in MPS I canines without adverse effects to clinical status, hematologic parameters, electrolytes, or transaminases. Two weeks following injection, we identified high levels of IDUA enzyme activity in necropsied synovium and more modest, but significantly elevated, IDUA activity levels in cartilage, serum, and liver. The IDUA activity levels correlated with degree of substrate clearance, with normalization of synovial HS and DS GAG and lysosomal histopathology scores; and reduction of cartilage HS and DS GAG, and normal, basophilic chondrocyte morphology. Although IDUA enzyme has higher *V*_max_ and lower *K*_m_ for DS [24], HS appears to be a more sensitive marker to treatment response, as it is largely absent from normal synovium and cartilage. Though KS and DS storage are more classically associated with articular pathology in MPS, HS storage does appear to contribute to arthropathy too, as it has proinflammatory properties [25, 26]. MPS type III patients also have some degree of skeletal dysplasia. In addition, HS and DS accumulation were found to impair BMP-4 signaling in multipotent adult progenitor cells derived from MPS I patients [27].

The presence of the IDUA transgene in synovium and liver at two-week postinjection prompts an interesting inference. Some amount of intra-articular delivered AAV9-*IDUA* vector likely escapes the joint space into the bloodstream to transduce hepatocytes and other body tissues, which lead to secretion of enzyme into the bloodstream. This suggests the potential utility of intra-articular gene replacement therapy to generate sufficient blood IDUA to treat other multisystemic MPS I pathologies; however, the study was not designed to assess effects of treatment upon either CNS or visceral disease manifestations.

The findings in the animals necropsied 52-week post-treatment identify challenges to an approach centered solely around intra-articular gene replacement therapy for MPS I. Animals treated at either 5E11 or 5E12 vg/joint doses continued to synthesize synovial IDUA enzyme, albeit slightly above wild-type levels. Shoulder cartilage IDUA in the high-dose +52 w animal was 33% of wild-type; strangely, the *IDUA* transgene was repeatedly observed only in chondrocytes of this animal but not in the synovium. This corresponded to some treatment effect, as shoulder and stifle synovial vacuolization were mild in the high-dose +52-week animal. Regardless, these subnormal/normal IDUA levels were unable to clear substrate; synovial and cartilage GAG levels in AAV9-*IDUA-*treated joints were equivalent to AAV9-*eGFP-*treated joints and markedly elevated compared to normal tissues. Cellular vacuolization was severe in chondrocytes of both animals.

Another important caveat to this proof-of-principle study is that MPS arthropathy affects all joints of the body; we focused gene replacement upon a small number of large joints in these animals. Safe gene replacement of every MPS-affected joint will be a challenge owing to the potentially large vector dosages required, and instead may need to focus upon joints at greatest risk for degeneration (hips) or joints with greatest impact upon daily function (shoulders, knees).

The presence of an anti-IDUA immune response may be one explanation for the lack of clinical efficacy in the older animals. The MPS I canine model is well known to develop a strong antibody response to either intravenously administered or AAV-transduced IDUA protein [28, 29]. Anti-IDUA antibodies are well described in both canine and murine MPS I models to inhibit enzymatic activity and divert enzyme trafficking to nonlysosomal compartments [29, 30]. Anti-IDUA antibodies were found in synovial fluid of both AAV9-*IDUA-* and AAV9-*eGFP-*treated joints, indicating that inhibitory antibodies can cross joint capsules. Alternatively, as some studies have shown, neonatally delivered transgene may become lost, especially in rapidly-dividing tissues [31]. In the two +52-week animals, IDUA enzyme was undetectable in serum and liver, which corresponded to a rapid increase in anti-IDUA serologies and concomitant diminution of IDUA enzyme in blood; IDUA transgene was absent from liver at necropsy. These factors are all potential etiologies for lack of clinical efficacy upon MPS I joint pathology in either +52-week animal.

Additional canine studies to explore the intra-articular route for MPS I therapy are planned; pups will be tolerized with a 5E12 vg/kg intravenous dosage of IV AAV8-*IDUA*, which has been documented to abrogate anti-*IDUA* immune response without cross-reactivity to subsequent AAV9-*IDUA* dosing [29]. As tolerized canines in this previous study expressed supraphysiologic CNS enzyme activity levels until the time of necropsy at 11 months of age, our follow-up studies will allow for assessment of sustained, high-level intra-articular enzyme synthesis with planned efficacy measures involving joint pathology utilizing ultrasound, magnetic resonance imaging, and other techniques [32]. Enzyme-linked immunosorbent spot assays will be performed to assess cellular immune response to both AAV capsid and IDUA protein following treatment. Concurrent intravenous and/or intracisternal *IDUA* gene replacement will also be explored to determine whether the other multisystemic manifestations of MPS I disease can be ameliorated. These studies will assess the feasibility of a multimodal, combination gene replacement therapy approach to address all manifestations of MPS I disease.

## Supplementary Material

Supple Mat Figure S1: synovial transgene and native *IDUA* PCRs. Figure S2: synovial lysosomal storage scoring. Figure S3: chondrocyte lysosomal storage scoring. (Supplementary Materials)

## Figures and Tables

**Table 1:** Absolute (% of wild-type) serum IDUA enzyme activity of the three animals treated in this study with respect to timing of treatment.

		Time pre-/post-intra-articular AAV9 treatment					
	Time of necropsy	−6 w	+2 w	+12 w	+24 w	+18 w	+52 w
High-dose 5E12 vg/joint	+2 w	0.00 (0%)	2.16 (49.5%)				
High-dose 5E12 vg/joint	+52 w	0.00 (0%)	n.m.	0.07 (1.6%)	0.11 (2.5%)	0.14 (3.2%)	0.00 (0%)
Low-dose 5E11 vg/joint	+52 w	0.00 (0%)	n.m.	0.00 (0%)	0.02 (0.46%)	0.05 (1.1%)	0.00 (0%)

All animals have undetectable serum enzyme at baseline (6 weeks prior to treatment); enzyme activities approach 50% of normal two weeks following treatment but decline afterwards, eventually returning to baseline at 52 weeks. Wild-type animal serum IDUA enzyme is 4.36 ± 1.31 units/mL. Data were generated from at least three independent experiments and shown as mean ± SD. n.m.: not measured.

**Table 2:** Comparison of baseline and postnecropsy synovial HS and DS levels in intra-articular AAV9-treated-MPS I canines.

	Baseline	Necropsy	Percent change
*Heparan sulfate*			
Synovial HS high-dose +2 w			
AAV9-*eGFP* shoulder	1.87	1.64	−12.3%
AAV9-*eGFP* stifle	3.01	3.78	25.6%
AAV9-*IDUA* shoulder	1.95	0.14	−92.8%
AAV9-*IDUA* stifle	4.11	0.04	−99.0%
Synovial HS high-dose +52 w			
AAV9-*eGFP* shoulder	0.83	5.14	519%
AAV9-*eGFP* stifle	4.5	19.4	331%
AAV9-*IDUA* shoulder	1.73	1.63	−5.8%
AAV9-*IDUA* stifle	2.79	1.25	−55.2%
Synovial HS low-dose +52 w			
AAV9-*eGFP* shoulder	1.27	4.35	243%
AAV9-*eGFP* stifle	1.17	9.59	720%
AAV9-*IDUA* shoulder	0.22	2.87	1205%
AAV9-*IDUA* stifle	2.16	3.17	46.8%
*Dermatan sulfate*			
Synovial DS high-dose +2 w			
AAV9-eGFP shoulder	6.64	4.1	−38.3%
AAV9-eGFP stifle	12.32	5.78	−53.1%
AAV9-IDUA shoulder	8.64	1.84	−78.7%
AAV9-IDUA stifle	12.5	0.8	−93.6%
Synovial DS high-dose +52 w			
AAV9-eGFP shoulder	3.52	9.72	176%
AAV9-eGFP stifle	10.13	13.1	29.3%
AAV9-IDUA shoulder	1.87	4.23	126%
AAV9-IDUA stifle	7.19	2.56	−64.4%
Synovial DS low-dose +52 w			
AAV9-eGFP shoulder	3.46	12.69	267%
AAV9-eGFP stifle	4.98	8.68	74.3%
AAV9-IDUA shoulder	0.43	4.55	958%
AAV9-IDUA stifle	4.33	3.81	−12.0%

**Table 3:** Comparison of baseline and post-necropsy cartilage HS and DS levels in intra-articular AAV9-treated-MPS I canines.

	Baseline	Necropsy	Percent change
*Heparan sulfate*			
Cartilage HS high-dose +2 w			
AAV9-*eGFP* shoulder	0.7	0.75	7.1%
AAV9-*eGFP* stifle	0.25	0.49	96.0%
AAV9-*IDUA* shoulder	0.72	0.41	−43.1%
AAV9-*IDUA* stifle	0.66	0.31	−53.0%
Cartilage HS high-dose +52 w			
AAV9-*eGFP* shoulder	0.35	9.01	2474%
AAV9-*eGFP* stifle	0.79	12.7	1508%
AAV9-*IDUA* shoulder	1.68	5.4	221%
AAV9-*IDUA* stifle	0.9	8.96	896%
Cartilage HS low-dose +52 w			
AAV9-*eGFP* shoulder	0.42	7.09	1588%
AAV9-*eGFP* stifle	1.26	7.33	482%
AAV9-*IDUA* shoulder	0.99	5.99	505%
AAV9-*IDUA* stifle	1.04	8.82	748%
*Dermatan sulfate*			
Cartilage DS high-dose +2 w			
AAV9-*eGFP* shoulder	2.58	3.3	27.9%
AAV9-*eGFP* stifle	3.56	4.11	15.4%
AAV9-*IDUA* shoulder	0.4	2.43	508%
AAV9-*IDUA* stifle	3.66	4.05	10.7%
Cartilage DS high-dose +52 w			
AAV9-*eGFP* shoulder	0.27	15.69	5711%
AAV9-*eGFP* stifle	2.36	21.09	794%
AAV9-*IDUA* shoulder	3.41	14.22	317%
AAV9-*IDUA* stifle	3.46	14.14	309%
Cartilage DS low-dose +52 w			
AAV9-*eGFP* shoulder	0.29	15.22	5148%
AAV9-eGFP stifle	4.24	13.64	222%
AAV9-*IDUA* shoulder	0.44	12.33	2702%
AAV9-*IDUA* stifle	3.14	17.5	457%

## Data Availability

The molecular, enzymatic assay, glycosaminoglycan quantitation, serology, and histopathology data used to support the findings of this study are available from the corresponding author upon reasonable request.
