# Supplementary material for: Intra-Articular AAV9 α-l-Iduronidase Gene Replacement in the Canine Model of Mucopolysaccharidosis Type I
Source: Adv Cell Gene Ther. Author manuscript; Available in PMC 2025 Aug 28. (PMC12382353; doi:10.1155/2023/7419017)
Supplement: Supple Mat — Figure S1: synovial transgene and native IDUA PCRs. Figure S2: synovial lysosomal storage scoring. Figure S3: chondrocyte lysosomal storage scoring. (Supplementary Materials) [file NIHMS2017112-supplement-Supple_Mat.docx]

**SUPPLEMENTARY MATERIALS**
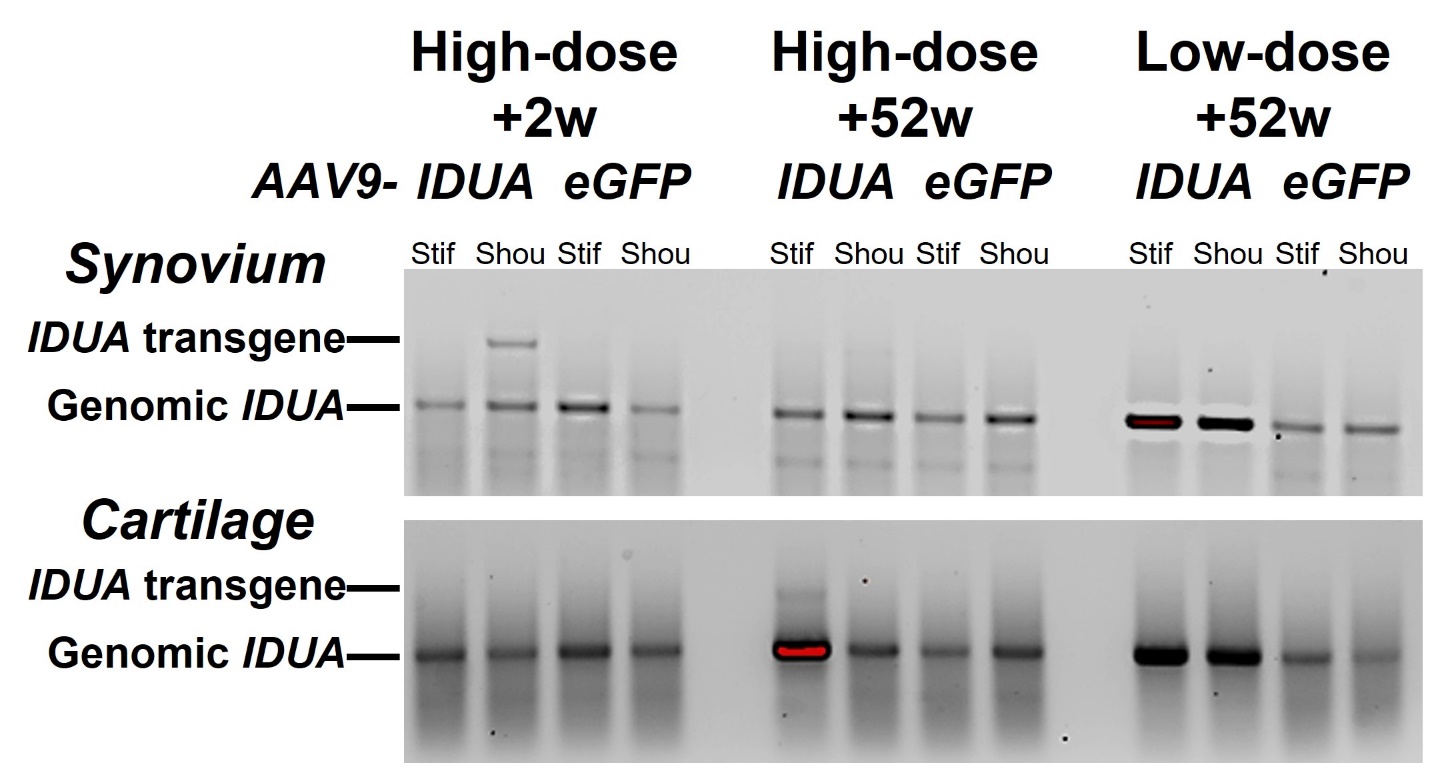


**Figure 1S**. **Synovial transgene and native *IDUA* PCRs**. All joints demonstrate presence of the genomic *IDUA* fragment; none of the AAV9-eGFP-treated joints demonstrate the *IDUA* transgene. In synovium, only the shoulder (and not the stifle) of the high-dose, +2w animal demonstrates presence of the *IDUA* transgene. Stif: Stifle; Shou: Shoulder.


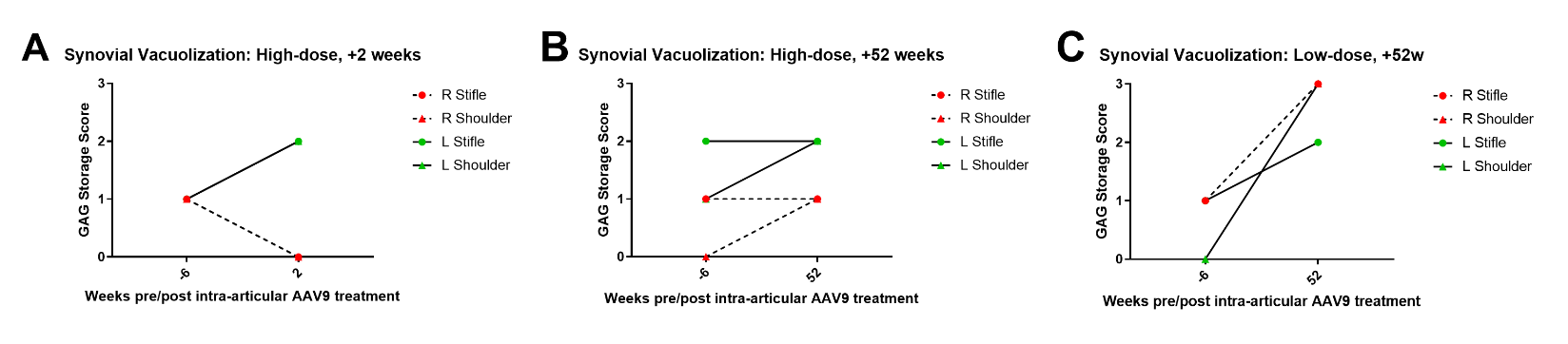


**Figure 2S. Synovial lysosomal storage scoring** (0, none; 1, mild; 2, moderate; 3, severe storage). Only in the high-dose, +2w animal (**A**) is there clearance of tissue storage at time of necropsy. There is mild lysosomal storage in **AAV9-*IDUA*** treated high-dose, +52w synovium (**B**), but development of severe lysosomal storage in **AAV9-*IDUA*** low-dose, +52w synovium (**C**).


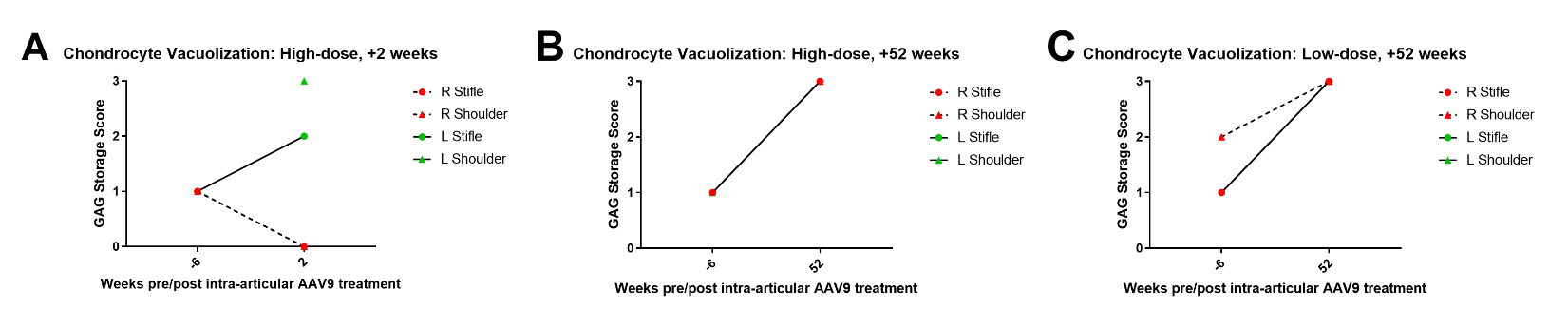


**Figure 3S. Chondrocyte lysosomal scoring**. (0, none; 1, mild; 2, moderate; 3, severe storage). Only in the high-dose, +2w animal (**A**) is there clearance of tissue storage at time of necropsy. There is severe lysosomal storage in both the high-dose, +52w (**B**) and low-dose, +52w (**C**) animals.
